# Self-Management and Self-Management Support Outcomes: A Systematic Review and Mixed Research Synthesis of Stakeholder Views

**DOI:** 10.1371/journal.pone.0130990

**Published:** 2015-07-10

**Authors:** Emma Boger, Jaimie Ellis, Sue Latter, Claire Foster, Anne Kennedy, Fiona Jones, Vicky Fenerty, Ian Kellar, Sara Demain

**Affiliations:** 1 Faculty of Health Sciences, University of Southampton, Southampton, United Kingdom; 2 Department of Social Care and Education, St George’s and Kingston University of London, London United Kingdom; 3 Institute of Psychological Sciences, University of Leeds, Leeds, United Kingdom; The University of Leeds, UNITED KINGDOM

## Abstract

**Introduction:**

Self-management has received growing attention as an effective approach for long-term condition management. Little is known about which outcomes of supported self-management are valued by patients, their families, health professionals and those who commission self-management services. This study systematically reviewed published empirical evidence in accordance with PRISMA guidelines to determine the outcomes of self-management valued by these key stakeholder groups, using three prominent exemplar conditions: colorectal cancer, diabetes and stroke.

**Aim:**

To systematically review the literature to identify which generic outcomes of self-management have been targeted and are considered important using three exemplar conditions (colorectal cancer, diabetes and stroke), which collectively have a range of features that are likely to be representative of generic self-management issues.

**Methods:**

Systematic searching of nine electronic databases was conducted in addition to hand searches of review articles. Abstracts were identified against inclusion criteria and appraised independently by two reviewers, using a critical appraisal tool. Synthesis of findings was conducted using mixed research synthesis.

**Results:**

Over 20,536 abstracts were screened. 41 studies which met the review criteria were fully retrieved and appraised. The majority of evidence related to diabetes. Few studies directly focussed on stakeholders’ views concerning desired self-management outcomes; the majority of evidence was derived from studies focusing upon the experience of self-management. The views of health care commissioners were absent from the literature. We identified that self-management outcomes embrace a range of indicators, from knowledge, skills, and bio-psychosocial markers of health through to positive social networks.

**Conclusions:**

Patients’, families’, health professionals’ and commissioners’ views regarding which outcomes of self-management are important have not been clearly elicited. The extent to which bio-psychosocial indicators relate to successful self-management from the perspectives of all groups of stakeholders is unknown. Further investigation regarding which self-management outcomes are considered important by all stakeholders is necessary to guide the commissioning and design of future self-management services.

## Introduction

Long-term conditions (LTC), or Chronic diseases, are health problems that require ongoing management over a period of years or decades [1]. LTCs are responsible for most of the disease and deaths in European countries [2], with the proportion of deaths due to LTCs projected to rise from 59% in 2002 to 69% in 2030 [3]. In the United States, approximately 85 per cent of healthcare spending is for people with long-term conditions [4]. In England, around 15 million people live with a LTC [5], accounting for 70 per cent of hospital and primary care budgets [6]. How health professionals, policy makers and providers should respond to the expansion in the number of people with LTCs is therefore a critical contemporary issue. Self-Management (SM) has been highlighted as one way of approaching this issue [7,8]. SM represents an ideological shift away from patients as ‘passive’ recipients of treatment to empowered individuals who are partners in the effective management of their health [9]. We view self-management from the perspective that the majority of people with LTCs manage their own health, without direct professional input for the vast majority of the time. They do this by performing a range of activities they consider to enhance their health and well-being. Such activities include but are not limited to behaviours directly recommended by health professionals e.g. taking medication; general health behaviours, such as keeping fit; and, activities to promote psychological well-being such as relaxation. From this perspective self-management also involves the tasks people do to navigate health services and to take an active part in their treatment planning such as shared decision-making. Differing degrees of support for self-management, from professionals and/or peers may be required at varying times.

The provision of support to enable patients and families to be confident and capable in managing health conditions underpins effective SM [9,10]. Various SM support interventions to assist people to optimise the management of health have been trialled [11]. These have taken a variety of forms including lay or professional led, generic or disease specific, group or individually delivered [11], and typically focus on chronic disease self-management education programmes [12,13] most often based on Social Cognition Theory [14,15]. The main objective of such interventions is to provide people with the information and skills that enhance their ability to manage their health; for example, communicating with health professionals, goal-setting and problem-solving. Several studies have shown that effective self-management can result in modest improvements in self-efficacy [16–18], mood [19–21], physical symptoms and function [22–24]and differing levels of reduced health service utilisation [9].

SM support interventions for LTCs use a diverse variety of outcome indicators to assess effectiveness. A recent review of research identified over 70 different potential variables used to evaluate self-management programmes [25]. Research tools that measure the impact of self-management interventions most frequently focus upon self-efficacy, health status, mood and quality of life [26]. However the conceptual relationship of such indicators to self-management has not been clearly articulated. Moreover, tools which purportedly measure the effectiveness of self-management interventions have been developed with a lack of patient input and poor methodological quality, raising questions about their validity and reliability [27]. The diversity of indicators targeting different purported outcomes is indicative of the conceptual complexity of SM. However, the uncertainty surrounding which outcomes of SM support are valued and by whom and in what context, represents a critical limitation in the design and evaluation of SM interventions [28]. For example, it is frequently assumed that improvements in clinical indicators and/or health behaviours are the outcomes of most value [29,25] yet there is little empirical evidence about which outcomes are most valued by patients, their families, health professionals and health service managers (commissioners*). Unless an understanding of the outcomes desired by all stakeholders is sought, there is a risk that SM support may not adequately reflect the priorities of each stakeholder. Indeed, this may account for the unacceptably high attrition rates from and low uptake of SM support interventions both in research and clinical practice [30,31], contributing to limited value for money.

A scope of the outcomes commonly targeted by self-management support interventions, and which are valued by stakeholders, is urgently needed to inform future practice and research. This systematic review and mixed research synthesis specifically aims to investigate which generic high level outcomes of self-management have been considered important by differing stakeholders thus far.

*In the UK context, commissioners are responsible for planning, agreeing and monitoring health services.

## Methods

A mixed-research synthesis was adopted. Mixed-research synthesis [32] allows both qualitative and quantitative findings, viewed as answering the same research questions, to be meaningfully summarised and synthesised so that understanding is broadened. Established PRISMA guidelines for conducting and reporting systematic reviews were followed [33,34]. Details of the protocol for this systematic review were registered on PROPSERO, (accessed at www.crd.york.ac.uk/PROSPERO/display_record.asp?ID=CRD42014.007316.)

### Scope of review

To focus the review, three exemplar conditions, where SM features as a major component and which reflect a range of factors relevant to SM, were selected: diabetes (type I and II), stroke and colorectal cancer. Each condition has markedly different disease trajectories, treatments and core care pathways and generates differing physical, social and cognitive issues. Consequently, SM foci vary in each: SM for stroke focuses upon recovery and adaptation [35,36]; for diabetes, on maintenance and prevention of deterioration [37,38]; and, in colorectal cancer on preventing recurrence or detecting possible recurrence early, building confidence to live with and manage consequences of cancer and its treatment and seeking help and support as required [39,40]. Secondly, the current provision of SM support is variable: SM support is typically more widespread for diabetes compared with stroke and colorectal cancer. Thirdly, the condition associated stigma, which may impact upon the success or otherwise of SM, may differ [41–43]. The heterogeneity between conditions and associated SM issues thus afforded a focus on generic SM outcomes, as opposed to condition specific SM outcomes.

### Search strategy

Published literature was systematically searched using the following electronic databases; Allied and Complimentary Medicine database (AMED), British Nursing Index (BNI), Cumulative Index to Nursing and Allied Health Literature (CINAHL), EMbase, Medline, Psychinfo, Web of science, in addition to hand searches of reference lists of review articles. A formal database search strategy using a combination of free text search terms and subject headings was created in consultation with an information scientist (VF). This is shown in S1 Fig. Boolean operators or Mesh terms, along with truncated terms (e.g. self-manag*) were used for search combinations of key terms (Table 1).

**Table 1:** Search combinations used for electronic databases

Self-management AND outcomes AND:	
Patients **AND**	Stroke OR, Diabetes OR, Colorectal cancer
Family/Carer **AND**	Stroke OR, Diabetes OR, Colorectal cancer
Health professionals **AND**	Stroke OR, Diabetes OR, Colorectal cancer
Commissioners **AND**	Stroke OR, Diabetes OR, Colorectal cancer

### Inclusion and exclusion criteria

Studies were restricted to English language reports. Since little is known about the preferences of stakeholder groups, and which study design these might be represented by, inclusion criteria were kept broad and were not excluded based on the length of time that a person had been living with the condition. Studies which arguably have relevance to self-management, for example, secondary prevention or shared decision making, but which did not use the terms self-manag* or self care* (S1 Fig) were not included.

Inclusion criteria:

- Empirical literature published 1995–June 2014
- Studies which specifically concerned self-management in relation to diabetes, stroke or colorectal cancer. Studies where treatment was completed and where treatment was ongoing were included.

Exclusion criteria:

- Study populations under 18 years of age
- Gestational diabetes.

### Study quality

Empirical evidence derived from any research design was included. Papers were not excluded on the basis of quality judgements; however, quality was rated to aid interpretation of findings. In the absence of a critical appraisal tool suitable for comparing quality across different study designs, we adapted a tool developed by reviewers at the Evidence for Policy and Practice Information centre [44,45] through team consensus (S2 Fig). Each study was assessed independently by two researchers (EB and JE) for bias/rigour in sampling, methods, data collection, analysis and reliability of findings plus the relevance of findings in relation to self-management outcomes. Each study was assigned a quality score out of a maximum of 25 (mixed methods designs) or 22 (either quantitative or qualitative only designs).

### Data Abstraction

**Fig 1:**
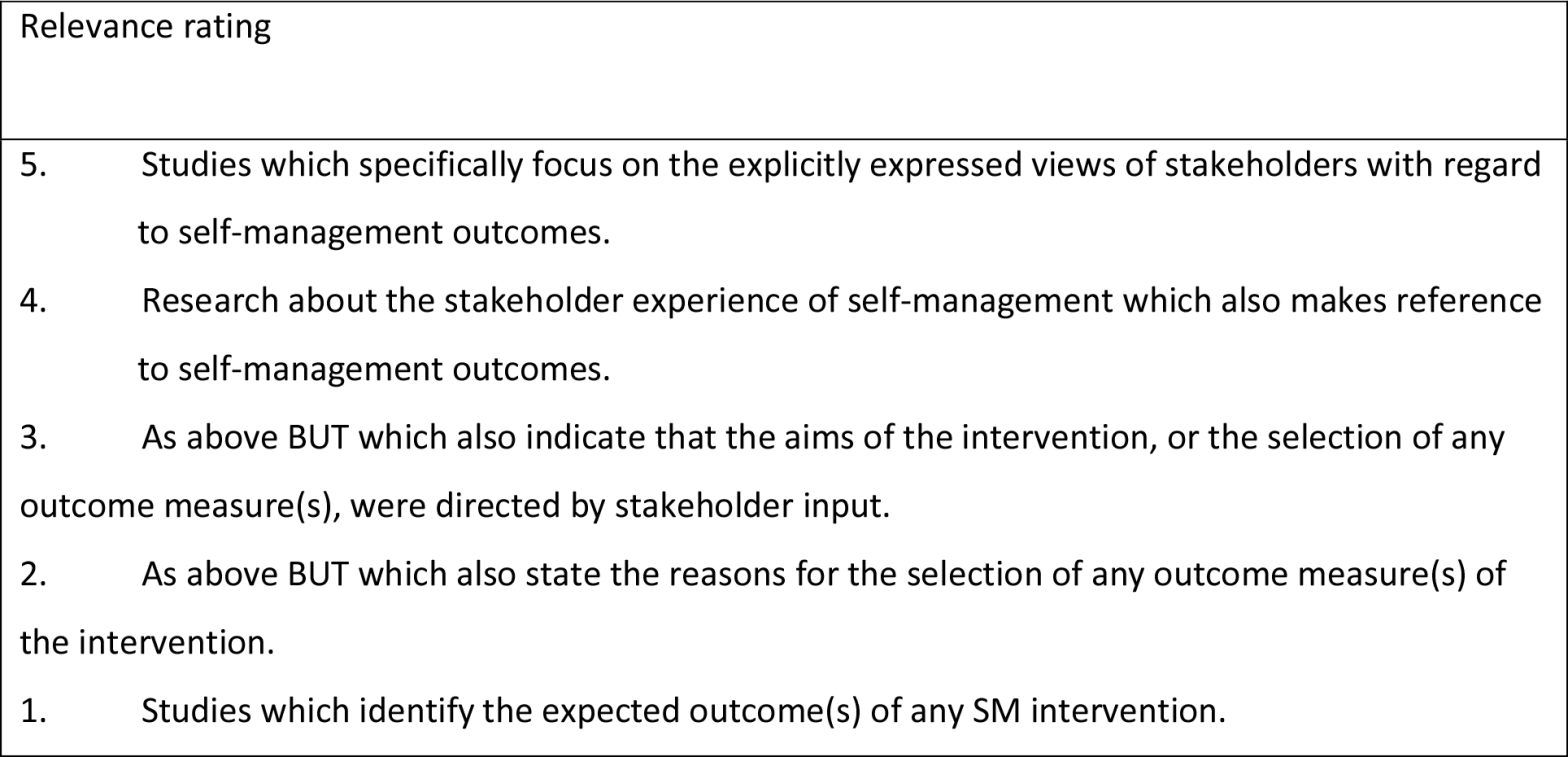
Relevance criteria for rating studies.

Titles and abstracts were independently screened for inclusion by two reviewers (EB & JE). Abstracts which met the inclusion criteria were rated in terms of relevance to the aims of the review on a 1–5 scale using pre-established criteria (Fig 1). Disagreements in ratings of abstracts were resolved through discussion, and review of the full paper if unresolved. Studies rated ≥ 3 points were retrieved for full appraisal, and independently double-rated for quality by EB & JE using the critical appraisal tool. Study design, setting, methods, participant characteristics and study findings were extracted using a standardised data extraction form. A random 25% of included articles were reviewed and rated for quality by a third reviewer (CF, FJ or IK) to confirm reliability and ensure rigour.

Management, synthesis and analysis of findings were conducted by EB and JE using NVivo 10 software for qualitative data analysis [46]. Qualitative excerpts or quantitative indicators relating to outcomes of self-management were independently extracted and relevance to self-management agreed by EB and JE. Data from qualitative sources were included if they consisted of direct quotations, which expressed desired outcomes of self-management and which were used as exemplars of reported themes. Using the excerpts, the expressed desire of self-management was summarised and entered as descriptive codes in NVIVO. Data from quantitative sources which reported the indicators explicitly used to indicate successful self-management were considered relevant and extracted. For example, where a study measured Quality of Life or reduction in blood glucose levels, as a purported outcome of an intervention, the outcomes ‘Quality of Life’ and ‘Reduction in Blood glucose’ was entered into NVIVO. All data were then analysed and synthesised using thematic analysis [32]. Extracted data were assigned codes. Each data extract was revisited and described using existing or new codes until all data could be described fully. Codes were then thematically synthesised to generate higher level outcomes. For example, the outcomes ‘feeling normal’ and ‘having choices and options’ were synthesised to become part of the theme ‘being me’. A third reviewer checked the analysis audit trail (SD).

## Results

**Fig 2:**
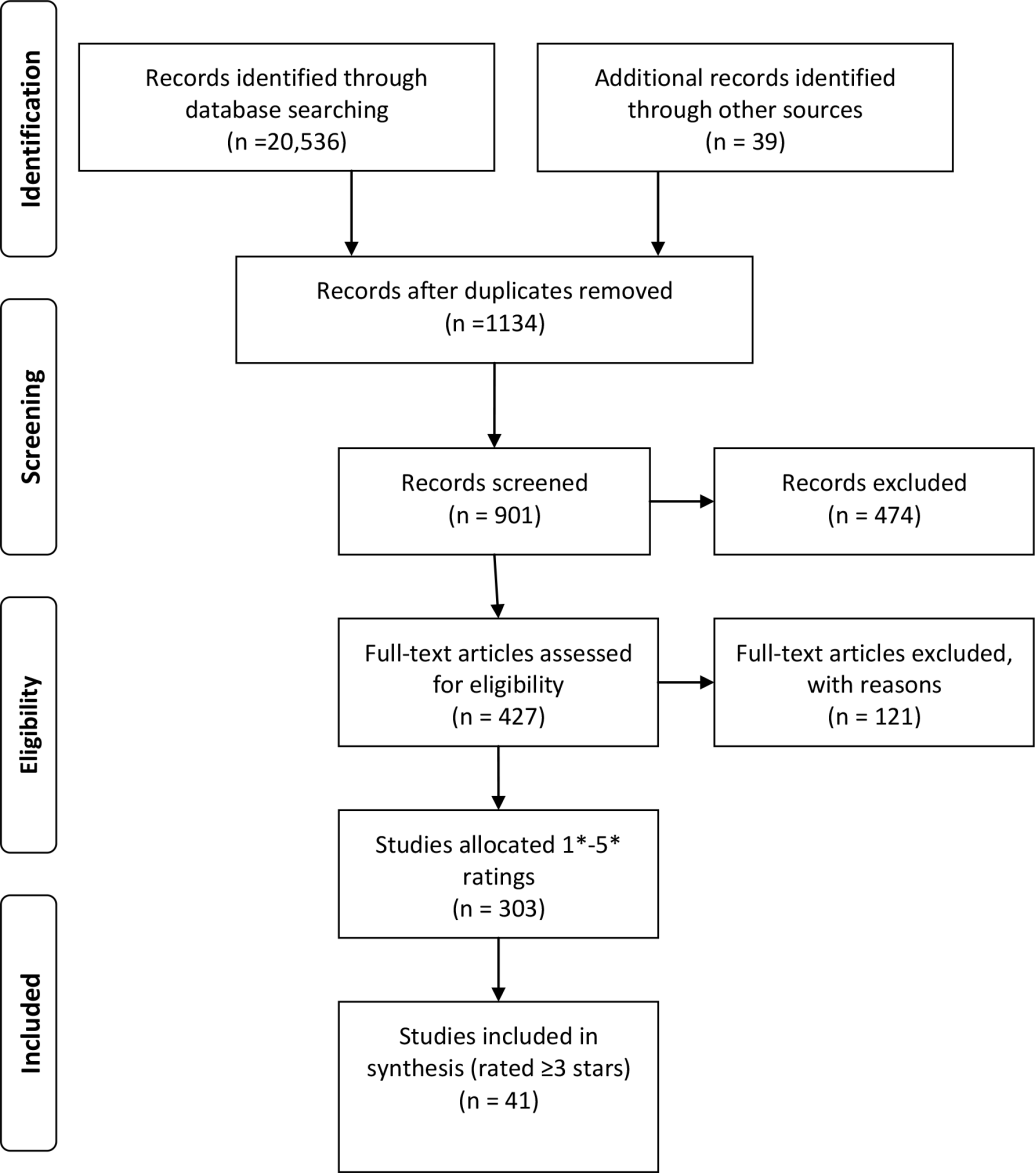
PRISMA Diagram.

20,536 citations were retrieved in total. After the removal of duplicates and non-English language papers, 901 abstracts were screened, those not meeting the inclusion criteria were excluded. Of 427 abstracts, 306 were relevant to the aims of the review (Fig 2). 41 abstracts (13.5%) were rated 3 stars or above. 40 were fully retrieved for appraisal and data extraction. 1 abstract could not be retrieved via library resources or via contact with authors [47]. Fig 2 depicts the PRISMA flow of data. The majority of evidence was generated from studies on diabetes (80%).

## Profile of included studies

**Fig 3:**
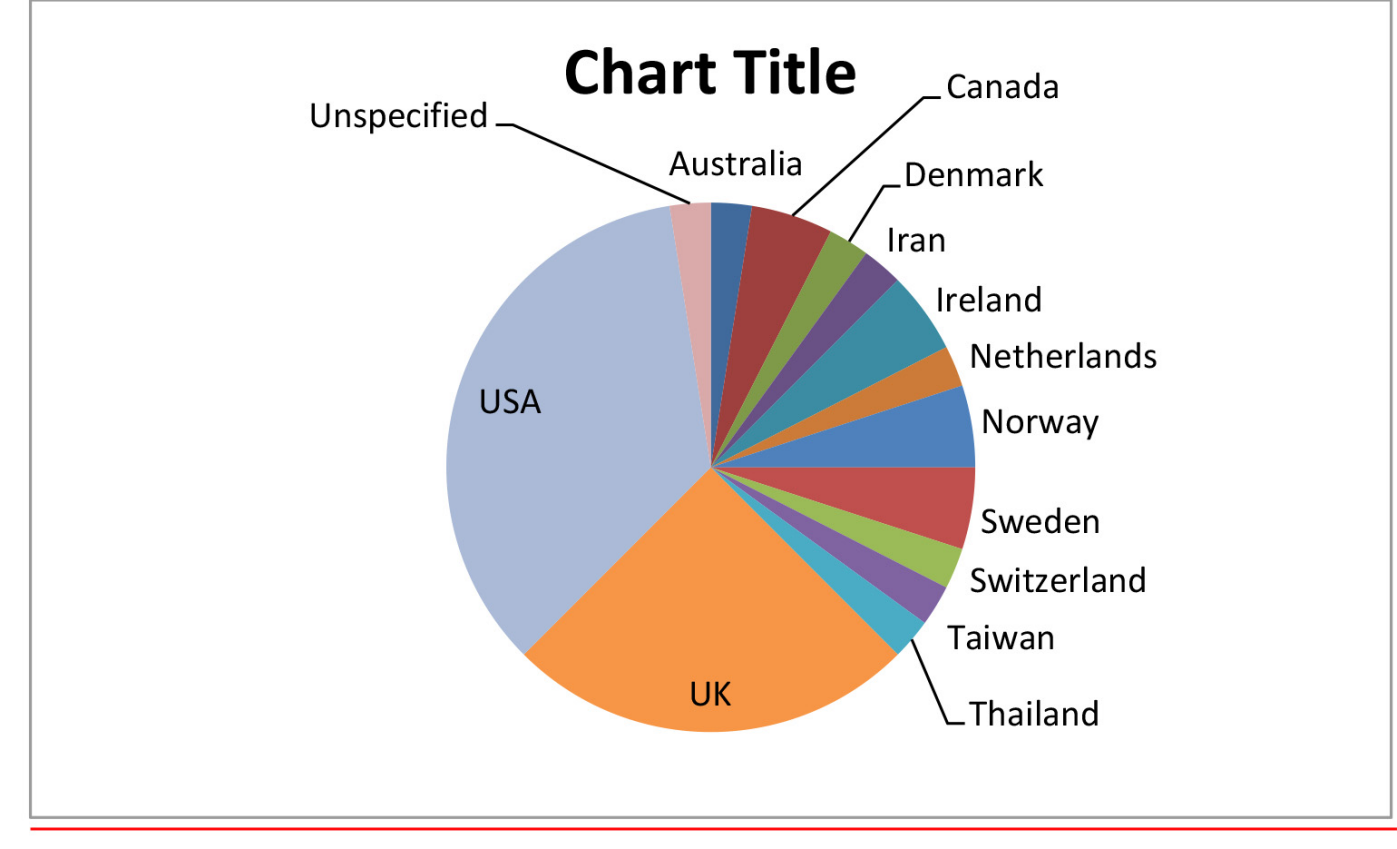
Origin of included studies (n = 40).

Those studies which scored ≥3 stars originated from 13 different countries, most predominantly the USA and UK (Fig 3). A variety of study designs were utilised by included studies, the majority of which were qualitative (31 studies, 77.5%) (Table 2). The more prevalent study designs were individual interviews (n = 18, 45%), focus groups (n = 10, 25%), quasi-experimental and survey (both n = 4, 10%).

**Table 2:** Quality ratings of included studies.

*Colorectal Cancer*			
Country & Setting (*Quantitative design) [reference]	Study design and sample	Mean quality rating (maximum score 22)	Extracted self-management outcomes
UK, Community [50]	Focus groups with colorectal cancer survivors (n = 40) **(P)**	14	Coping with fatigue—Coping with fear of recurrence—Coping with Sexual dysfunction—Improving mobility—Coping with bowel symptoms—Wanting clarity of information-Returning to previous self
UK, Cancer centre [67]	Longitudinal Qualitative design. Individual interviews pre and 6mths post chemotherapy treatment (n = 11) **(P)**	10.5	Managing symptoms—Being resilient for treatment- Not letting treatment/side effects interfere with life—Prevent reoccurrence—Maintain health-To be in control
UK, Cancer centre [60]	Individual interviews pre and 6mths post chemotherapy treatment (n = 11) **(P)**	10.5	Manage treatment/symptoms—Prevent complications-To be as fit as possible—Maintain a sense of ‘normality’
USA, Cancer clinics [62]	Semi-structure interviews with cancer free adults, treated for Colorec Ca in the pre 0–24 months (n = 41) **(P)**	12	Gain and maintain general fitness-Reduce risk of reoccurrence—Improve chances of recovery—Prevent/ stop complications-Control pain—To return to pre-cancer life-To address lifestyle issues (diet, smoking, weight, exercise)
***Diabetes***			
UK, 4 universities [92]	Semi-structured interviews and diaries. University students (n = 17, aged 18–25yrs) with T1DM **(P)**	9	Regulate blood sugar—Reduce risk of complications—Take part in social activities without complications—Feel good about selves—Feeling normal
USA, Health centre [71]	2 FGs with HCPs—nurses, educators, physicians, paraprofessionals, outreach workers, nurse practitioners, physicians assistants (n = 15) 4 FGs with Latino adults 30–79 yrs old with T2DM (n = 37) **(HCP**, **P)**	12	Diet and exercise to control blood sugar **(HCP)**–Control blood pressure **(HCP)**—To improve quality of life **(P)**—Prevent deterioration of condition **(P)**—Stay healthy **(P)**
Ireland, GP practices and hospital diabetic clinics [93]	In-depth open ended interviews. Adults with type I or II DM (n = 17) **(P)**	12	Control blood sugar—Maintain a healthy weight
UK, GP surgeries [94]*	RCT. Video SM inter v. control group >18 yrs, newly diagnosed DM within previous 6/12s (n = 42) **(HCP)**	10	HbA1c—Body weight- Lipid profile—Improve quality of life- Improve dietary intake-Improve physical activity
USA, General internal medicine and endocrinology clinics of University hospital [68]	Secondary analysis of in-depth interviews Older (>65yrs) adults with T2DM (n = 28) **(P)**	7	To be able to walk—Maintain independence—Prevent complications/slow deterioration—Control blood glucose level—To feel good
UK, Diabetes Education network database [95]	Interviews with specialist nurses and dieticians (n = 5) **(HCP)**	10	Prevent complications—To feel competent-To feel confident—Improve mental and emotional wellbeing—Improve quality of Life—HbA1C
Sweden, Primary Health centre [66]	Structured conversation. T2DM adults (at least 1 year, 55–75yrs), & who ‘followed a diet and tablet or insulin regime’ (n = 8) **(P)**	3	Maintain stable blood sugar level—Regulate diet and exercise—Knowledge about blood glucose management
Canada, Community health centres and diabetes education centres [96]	Semi-structured interviews. T2DM English speaking adults (>18yrs) not using insulin, who monitored their BG levels, and self-identified as being of Black Caribbean or South Asian ethnicity **(P)**	12	Control blood sugar level—Prevent complications
USA, Community health centres (CHCs) [97]*	Quasi-experimental. Non-randomised evaluation of impact of community health workers on Diabetes SM v. non- participating CHCs **(HCP)**	10.5	Control blood sugar—Maintain biomedical markers (HbA1c, lipid profile, blood pressure)—To keep appointments—To have a healthy diet—To monitor blood glucose-To exercise
USA, Community [63]	Semi-structured interviews. African- American (n = 20) & Latino adults (n = 20) (38–72 yrs) with DM and had completed or were active in a Community Health Worker-led diabetes self-management program **(P)**	12	Control blood sugar—Prevent complications—Build confidence—To live longer and more healthily—Have hope—To enhance emotional support
Canada, Rural community-based chronic disease management Program [58]	Exploratory qualitative study. Interviews with DM patients who had received health coaching for ≥6 sessions (n = 3) **(P)**	5.3	To be healthy-To increase life expectancy—Lose weight—Feel in control of condition—To have good mood—To manage independently from HCPs
USA, City medical centre clinics [57]	In-depth interviews. Grounded theory approach >65yrs, T2DM plus 1 additional risk factor (n = 28) **(P)**	11	Maintain independence—Lose weight—Prevent complications—Control sugar levels—Stay healthy- Remaining independent—Staying alive
USA, Public health clinics [48]	Exploratory descriptive design. Interviews with adult Mexican-Americans with T2DM (n = 51). Interviews with HCPs from public health clinics and community health centres (n = 36) **(P)**	10	Control of blood sugars **(HCP)**—To have long-term health **(HCP)**—To have appropriate helpful information **(P)**—To manage blood sugars **(P)**- To looking after yourself to the best of your ability **(P)**
Taiwan, 3 teaching hospitals [69]	Focus groups (n = 5). Adults >20yrs, with T2DM, for >5yrs, (n = 41) **(P)**	11	To ‘cure’ diabetes-Control sugar levels-Achieve a balanced life (social-emotional wellbeing)—To consult professionals—To live a healthy life
Thailand, Urban communities [65]	Semi-structured. 1:1 interviews conducted in Thai. Thai Buddhist adults (>20yrs) with T2DM, able to read and write (n = 30) **(P)**	12.5	Control blood sugar—Maintain health—Prevent complications
USA, City clinics that serve the uninsured and under-insured [51]	Focus groups (n = 12). >18 yrs, English or Spanish speaking African American & Mexican Americans T2DM (n = 84) **(P)**	11	Lose weight—Avoid complications—Reduce health care costs—Manage sugar levels- To feel good—Maintain physical function- Reduce stress- Have control over treatment
USA, Urban medical university [75]	Focus Group (n = 6). Black women with T2DM (n = 7) **(P)**	6	Prevent complications-Improve Knowledge-Avoid deterioration—Not be reliant on poor professional knowledge
Denmark, RCT study population [64]	Focus groups (n = 7). T1 or T2DM (30–72yrs) who had participated in a 4 day SMI, or were about to participate in the SMI (n = 22) **(P)**	11.6	Control blood glucose level—Prevent complications—To feel confident-To fully participate in ‘normal’ life—Ability to manage condition yourself—A family that supports dietary changes
USA, Outpatient clinics [73]	Qualitative in-depth interviews. T2DM, >55yrs, having hypertension plus one other co-morbidity (n = 24) **(P)**	8	Maintain physical function-To feel good—To maintain independence—Maintain health—To live longer-To be able to take part in social activities-Lose weight—Maintain blood sugar levels-Improve diet & exercise-Avoid complications
Ireland, 5 DafNE study sites [98]	Interviews, Adults (>20yrs) with T1DM, a range of time since diagnosis, age and gender (n = 40). **(P)**	11.5	Avoid hypoglycaemia—Reduce worry/stress—Prevent complications- Gain knowledge-Improving HBa1C
USA, Community [49]*	Quasi-experimental DSM educational intervention v. conventional DSMI T2DM for at least 1yr, >40yrs (n = 33) **(HCP & P)**	8	Prevent complications **(HCP)**—Prevent deterioration of health **(HCP)**—Increase diabetes knowledge **(HCP)**—Psychosocial adaption to diabetes **(HCP)**—Increase empowerment **(HCP)**—Increase self-care activities **(HCP)**—Learn how to follow the self-care recommendations **(P)**—Prevent & deal with complications **(P)**
Scotland, 16 general practices and 3 hospital clinics, in 4 local health cooperatives [99]	Longitudinal interview study (time 2 interview 6/12s later) T2DM diagnosed within the previous 6 months (n = 40) **(P)**	11.5	Manage sugar levels—Prevent complications—Manage independent of Health professionals—Knowledge to act upon blood glucose readings
Switzerland, Outpatient clinics and GP practices [100]*	Self-report Questionnaire. Importance of 16 treatment goals rated on a scale plus level of importance participants perceived their HCP also attributed to goals T1 (n = 297)or T2DM (n = 205), German speaking **(HCP & P)**	11	Control blood sugar levels (**HCP)-**Quality of life **(HCP)**—Reduce weight **(HCP)**—Reduce daily hassles **(HCP)**—Develop treatment goals **(HCP)**—Reduce frequency of hypos **(HCP)**—High quality of life **(P)**—Weight reduction/maintenance **(P)-**Avoidance of daily hassles **(P)**—Gain good medical care and knowledge **(P)**
USA, 3 rural counties [74]	In-depth interviews (n = 63) using semi-structured guide. African American, American Indian and white Adults (>60yrs) with DM (1 or 2) for at least 2 yrs. **(P)**	11.5	Avoid complications (amputations, coma, blindness)—Control blood sugar levels- Avoid hypoglycaemia
Australia, Inner city, university hospital outpatient clinic and a support group website [101]	Semi-structured interviewsEnglish speaking young adults (18–38yrs) with T1DM (n = 20) **(P)**	10	Control glycaemic changes- Minimise risks associated with fluctuating BG—Improve Knowledge—Avoid complications
Norway, 2 Hospital Trusts [59]	Semi-structured interviews and Focus groups (n = 2) Adults, T2DM, who’d been to GP in past 3 yrs (n = 23) and who’d attended educational group programs **(P)**	11	Live a ‘normal’ life—Avoid complications—Lose weight—Have more energy—Increase well-being
Norway, Hospital Trust [102]	Semi-structured interviews Adults with T2DM about to undergo DSMI (n = 22) **(P)**	8.6	Maintain a balanced diet—Stabilise blood glucose—Manage side effects of medications—To be more relaxed—Manage/ prevent complications—Lose weight—Physical function–Knowledge—Gain reassurance—Be more active
Iran, Clinic (unspecified) [55]	Focus groups (n = 6) Adults withT2DM >6/12s (n = 43) **(P)**	14.5	Control blood sugar levels—Avoid complications—To be fit in order to care for family—To fulfil religious obligations—To be able to access to equipment—To have knowledgeable health professionals
UK, Community urban and rural areas [53]	Focus groups Adults with T2DM (2 FGs with newly diagnosed, 2 FGs with new oral therapy, 2 FGs with new insulin therapy) **(P)**	11	Manage diet—Emotional and social wellbeing—Lose weight—Professionals that are motivational and proactive—To gain knowledge
Not specified [54]	‘Think aloud’ technique– 3 sessions where all thoughts, decisions and impressions related to DM over a 1 week period were recorded (self). Adult experts in T1DM decision-making (diagnosed for ≥15 yrs) (n = 22)**(P)**	11.3	Control blood glucose levels- Avoid complications—Prevent hypoglycaemia and hyperglycaemia—Ability to have a good quality of life—To develop skills to manage diabetes
Sweden, Unspecified setting [103]	Content analysis of 3 open-ended questions, 12, 24 and 3–7 yrs following participation in a DSMI T2DM participated in a year-long experience based group education program (n = 139)**(P)**	9.5	Manage blood glucose- Avoid going onto medications—Avoid complications—Prevent worsening of condition—Maintain a healthy weight—Get satisfying support from professionals
USA, Urban community health clinic [72]	Focus groups (n = 6) Latino adults with T2DM 18–70yrs (n = 20) & their caregivers (n = 20) **(P) (F)**	10.5	Prevent complications—Reduce stress—To gain glycaemic control—Avoid complications—Develop coping strategies
***Stroke***			
UK, SM training programme for health professionals [70]	Case reflections. In-depth case reflections (n = 60) of therapists and nurses working in inter-professional stroke teams across the UK **(HCP)**	10	Self-efficacy—Achieve goals which are important to the patient
UK, Database of therapists trained in a SM approach [61]	Semi-structured interviews. Therapists trained in a SM approach (n = 11) and working in stroke in acute, community & rehab settings **(HCP)**	12.6	Make impairments better—Achieve good long-term health—Achieve goals which are important to the patient
Netherlands, Community [56]	Focus groups. Stroke survivors > 3/12s post-stroke, living in the community and discharged following rehab (n = 16) **(P)**	11	To recover (to be previous self)—Adjust to impairment—Gain help and support—Manage mood and emotions
USA, Two rehabilitation hospitals [52]*	Survey. Survey of self-care needs of stroke survivors from perspectives of family members (n = 166)**(F)**	6	Prevent falls—Stay active—Manage stress levels—Deal with emotional and mood changes—To increase dexterity, memory and function—Prevent complications—Improve communication—Maintaining adequate nutrition—Manage roles and relationships—Understand stroke—Deal with behaviour and personality changes—Learn about exercise/activity/rest

* **(P)**- Patient, **(F)**- Family and Friends **(HCP)** Health Care Professional

The mean quality score for the 40 included papers was 10.26 (range 3–14). Inter-rater reliability for quality ratings was strong at 0.742 (Pearson’s’ r). No paper explicitly aimed to seek stakeholders’ views on valued SM outcomes. In quantitative papers preferred outcomes were inferred from indicators used to measure the effectiveness of interventions: in qualitative papers, they were discernible from participant’s perspectives on and experiences of SM and SM support more generally. Studies rated at ≥3 stars provided data from the perspectives of patients (n = 1620), health professionals (n = 1559), and family members (n = 44) (Table 3). Commissioners’ views were not represented.

**Table 3:** Summary of the stakeholder perspectives captured.

	Patients	Health Care Professional	Family and Friends
Colorectal cancer	92	0	0
Diabetes	1512	1488	20
Stroke	16	71	24
**Total**	**1620**	**1559**	**44**

## Self-management outcomes

Six broad themes were identified which together describe the self-management outcomes in use expressed in the included papers; applicable knowledge, independence, positive network, being me, self-management skills and attributes and optimal bio-psychosocial health. No outcome can be claimed to be more important than the others. Each theme is next described and evidenced using exemplar data extracts. Quotation sources have been identified in parentheses using the information provided in the primary source; where this is absent it is because it was not reported upon in the primary source. Table 4. summarises the outcomes of self-management identified from the review, and indicates areas of overlap between stakeholder groups.

**Table 4:** Summary of self-management outcomes identified as important by stakeholder group.

Theme		Outcome	Patient	Family and Friends	Health professional
Applicable Knowledge		Condition knowledge (for patients)	✓	✓	✓
		Having trustworthy and accessible information and resources	✓		
Independence		Physical Independence	✓		
		Feeling in control of condition and having confidence to manage it	✓		
		Independence from health professionals	✓		
		Equity of power with professionals	✓		
		Feeling holistically supported by health services	✓		
		Positive relationships with professionals	✓		
		Involving family members in SM			✓
		Not being a burden to family	✓		
Being Me		Feeling 'normal'	✓		
		Maintaining social identity	✓		
		Managing condition within the context of my life	✓		
		Having choices and options over management strategies	✓		
SM Skills		Managing consequences of treatment	✓		
		Managing emotions		✓	
		Managing stress	✓	✓	
		Patients who are motivated to SM			✓
		Patients who are empowered			✓
Optimal Health	Emotional	Improved confidence / self-efficacy			✓
		Feeling good and well	✓		
		Improved patient quality of life			✓
	Physical	Improved health	✓	✓	
		Improved biomedical markers			✓
		Preventing deterioration	✓	✓	✓
		Staying alive	✓		
	Social	Meeting family expectations and being ‘useful’ to family.	✓		
		Improved relationships with family member with LTC		✓	
		Improved communication with family member with LTC		✓	

### 1. Applicable knowledge

There was evidence that gaining knowledge was a self-management outcome of importance to patients (colorectal cancer and diabetes), family members (stroke) and health professionals (diabetes). There were, however, differences in how knowledge was conceptualised. Diabetes health professionals advocated knowledge that was focused around the diabetes disease process and prevention of deterioration:

*‘‘I would like them (patients) to have their own monitoring systems*, *we try to get education classes*. *The problem is they haven't been taught how to buy healthy food”*

(health care practitioner [48].

In contrast, patients with diabetes and colorectal cancer wanted personally relevant knowledge that was applicable and sensitive to their personal situation. Patients often received knowledge, but found it difficult to access or apply, or irrelevant to their cultural identity:

*‘‘I know what to do*, *but not how*, *it’s not realistic”*

(diabetes [49])

*‘‘People (health professionals) give you such broad useless information…but there must be some parameters in which I could work”*

(female, colorectal cancer [50])

*‘‘ I have tried to get it (medicine) on the phone*, *but they never answer*… *a machine answers in English and I don’t understand anything”*.

(female, diabetes [51])

The bulk of the evidence for this theme was generated from the patients’ perspective. One quantitative study concerning family members’ perceptions of self-care needs following stroke [52] identified ‘understanding stroke’ and ‘learning about exercise’ as two priorities and forms of knowledge to target in self-management support. Some patients identified that knowledge needs to originate from trust-worthy sources, such as health professionals who could provide knowledge as a source of reassurance:

*‘‘I think continuity is important*, *seeing the same people (health professionals) and not get different advice from different people”*

(male patient, T2 diabetes[53])

Conversely, some research reflected patient’s views that health systems and professionals were not the primary source of knowledge, preferring to trust their own knowledge of their bodies and diabetes:

*‘‘I believed in the medical system- stupid me*. *There was so much concentration on the importance of tests that you didn’t need to pay any attention and you ignored what your body was telling you because the tests told you something else*.*”*

(Patient, T1 diabetes [54])

*“We don’t know to whom we can trust*. *Every doctor says something* (different)*”*

(Patient, T2 diabetes [55])

Based on the included papers, condition knowledge appeared to be a key outcome to target in self-management support, however patients and families wanted different forms of knowledge, tailored to their situations, compared to a perceived emphasis by health professionals on disease specific knowledge.

### 2. Independence

Family members’ perspectives on independence were not evidenced. Common to all three conditions, patients viewed independence as a desired outcome of self-management. For patients, this meant both independence over the areas of health and well-being important to them, and which they felt confident to manage, *and* being independent from others:

*‘‘ My husband says 'come let me do it*!*'…I always have to explain that I want to do that myself”*

(Female stroke patient [56]).

Physical independence, in particular to avoid being a burden to family members or dependent on health professionals, was also an important aim for patients.

*‘‘That is my goal*, *to remain independent*, *and of course*, *I do not ever want to become a burden to my sons”*

(T2 diabetes patient [57])

*‘‘That gives me control*, *and that's what I was striving for*, *I don't want to be reliant on a nurse to control my blood sugar level”*

(female T2 diabetes patient [58]).

Independence seemed to relate to having control over and responsibility for managing the condition. However who had ultimate responsibility for control of the condition was not always made explicitly clear for patients.

*‘‘I thought my doctor had the responsibility for all this (diabetes)*. *But now I understand that it’s me*. *I have to make the decisions myself*, *and I feel that I have taken control”*

(Female aged 50–59 years, T2 diabetes [59]).

*‘‘You do have a level of control and I think whatever you do…there are things that you can always do yourself to help”*

(patient, colorectal cancer, [60]).

In stroke care, there was evidence that health professionals also viewed patient independence, in terms of the recovery goals they sought, as an important self-management outcome. Stroke health professionals acknowledged that self-management support could enable patients to have more independence and feel more in control: *‘‘empowering them* (patients) *to maximise their potential from within themselves”* (Stroke Physiotherapist, [61]). Descriptions of what empowerment meant in the context of having experienced a stroke were absent, however stroke professionals viewed patient empowerment as central to self-management: *‘‘*(self-management is) *the patient taking ownership for guiding their therapy”*(Stroke Occupational Therapist, [61]).

‘Empowerment’ was identified as an important outcome indicator of a self-management education intervention co-created with people with diabetes [49] suggesting empowerment is of importance to patients too. Independence is an important outcome for patients and health professionals, and one that could be a focus for future interventions.

### 3. Positive network

From the perspectives of patients, a positive social network that contributed towards an individuals’ ability to manage their health and well-being was both an important outcome of self-management, and a factor important to enabling successful self-management. The perspectives of health professionals and families were not evidenced in the selected papers. Two clear contributors to a positive network were identified; informal relationships and health professionals and services.

#### Informal relationships

Support from other people was considered an important outcome of self-management support across all health conditions. Perhaps unsurprisingly, people with colorectal cancer and diabetes who took part in group self-management interventions wanted support from people with a shared experience of the condition *‘‘It’s another thing to talk to someone who has gone through the treatment”* (Hispanic male, 54 years old, stage III colorectal cancer, [62]). ‘*‘It’s been really exciting going on* (group self-management education programme)…*it's been very lonely*” (patient diabetes, [63]). People with stroke and diabetes also drew on the experience of family members. The exerts below suggest that self-management in these conditions may be something co-created with family members both because of the potential impact on the whole family and the role of family members in supporting, planning and reminding relatives of activities important to self-management.

*‘‘My wife is my manager*, *self-management is something you do together*.*”*

(male stroke patient, [56]).

*‘‘It is best to be united in taking care of the diabetes*. *Take supper*, *for instance*. *My wife*, *she’s on low-calorie diet*, *so we actually have to eat the same thing*. *I think it’s easier when we are two*.*”*

(male diabetes patient, [64]).

#### Relationships with health professionals and services

Evidence from the selected papers suggests that the relationship between patients and health professionals is fundamental to ensuring professional support for self-management is effective. Patients, across all conditions, expressed a need to be treated as individuals by health professionals: *‘‘I need to feel my doctor is interested in my health*, *I need to feel I can talk to him and he is trying to understand me”* (diabetes patient, [63]). People with diabetes and colorectal cancer reported wanting holistic advice from their health professionals that did not focus only on biomedical information and which was relevant to their situation:

*‘‘your diet*, *your lifestyle*, *how much exercise you’re getting*, *…and also the other aspects which doesn’t really get enough recognition is stress and anxiety…*.*I would like to see a package of those things”*

(female colorectal cancer patient, [50]).

*‘‘My doctor is maybe 90% medicine and very little information of what to do”*

(diabetes patient, [63]).

Within diabetes, where lifestyle-related behaviours are particularly important to management, patients often sought to create a ‘good impression’ and broker a positive relationship by appearing to follow health professionals’ recommendations. Some patients wanted to fit the ideal of the "perfect patient," one who conforms to the "ideal standards," even if this meant concealing behaviour:

*‘‘When I have eaten a lot of sweet fruits*, *for example almost a kilo(gram)*, *I try not to eat anything one week after*, *especially before I see the doctor for a blood test”*.

(Male patient, T2 diabetes [65]).

*‘‘ I would take the insulin with me and went out to a pub or something and have a beer with dinner… Sometimes I’d be having low blood sugars or high blood sugars and it was a hit and miss sort of thing*. *But I didn’t tell anybody*. *I didn’t tell my nurses that I was doing that because they had said this is how many units of insulin you need to be taking”*

(T1 diabetes patient,[54]).

Patients in all three conditions reported that being able to access health professional advice and resources that enable self-management when needed was key to feeling supported. For people with stroke and colorectal cancer, their accounts often centred around feeling abandoned and not ready to manage by themselves once acute or formal services ended: *‘‘I missed* [having] *somebody to guide me after discharge—to stay active*, *to direct me”* (Male stroke patient [56]). In contrast, people with diabetes appeared to have more regular access to health professional support which contributed to people feeling supported: ‘*‘She* (nurse practitioner) *is usually very accessible and I feel strong support from her”*. (Diabetes patient, [66]).

Both formal and informal elements of support appeared to contribute to patients feeling enabled to self-manage and were important outcomes for self-management interventions from the perspectives of patients and professionals. Recognition is needed that developing positive support networks is a process necessary to aid self-management.

#### 4. Being me

Patients from all three conditions expressed a sense that their respective health conditions should not define nor dictate their lives. There were slight differences between conditions. Following colorectal cancer and stroke, people often talked in terms of regaining their pre-diagnosis lives: ‘*‘trying to be as normal as possible and trying to just be you*’ (Colorectal cancer patient [67]).

*‘‘keep going and be the person I always was*”

(Female stroke patient [56]).

In contrast, although people with diabetes expressed a strong desire to ‘‘*be normal”* (Patient with T2 diabetes [68]), this was frequently challenged by the burden associated with trying to manage blood glucose control: ‘‘*I feel completely outside when the others are sitting with a piece of cake*. . .” (female diabetes patient [64]). People often found ways to manage their condition in the context of their everyday lives. This included adapting tasks to suit the demands of their condition and integrating management into a usual routine ‘*‘It is heavy work to clean the whole house*. *But I can do a part of it*. *Yes*, *and every time I try a bit further*” (female stroke patient [56]), ‘*‘It’s* (diabetes) *part of your life*! *This is self-management*! *I always integrate diet and exercise into my daily life*” (patient, T2 diabetes [69]). People expressed a need for help and advice on managing their condition in a way that did not compromise important additional aspects of their lives, or force them to make difficult decisions. For example, financial constraints were often a concern: *‘‘Test strips are very expensive*. *On the other hand*, *doctors recommend us to check our blood glucose 3–4 times a day*. *But we cannot afford it*. *So we are obliged to check it every other week”* (patient, T2 diabetes [55]). Unless people could self-manage in a way that fitted their lives, advice would be unlikely to be adopted, ‘*‘You can tell me all day to do something*, *but it’s not going to help if I don’t know how to do it*.*”* (patient, diabetes [63]).

#### 5. Self-management skills

There was evidence from health professionals, patients and family members of people that a positive outcome of self-management support would be for patients to develop the skills or attributes necessary to assist management of health and well-being. Diabetes health professionals felt that patient motivation for self-management was a key requisite of success. Goal-setting was also cited by stroke and diabetes professionals as a key self-management skill [70, 71]. In contrast, patients and family members did not identify motivation or goal-setting as important self-management outcomes.

Patients from all conditions identified the need to develop skills in managing emotions and stress in order to maintain health and well-being.

*‘‘The physical pain is bearable*, *but the emotions and grief*. . . *there are no ‘emo-tablets*! *“‘*

(stroke patient [56])

*‘‘They don't give you nothing for stress they never talk to you about stress*.*”*

(Female T2 diabetes patient, [72])

*‘‘Personally I would like to see some sort of survivorship audit where they’re looking at…*. *and also stress and anxiety … which is as much a side-effect as diarrhoea and neuropathy”*

(Female colorectal cancer patient [50]).

Family members of stroke patients also wanted support in helping their relation to manage stress and emotions [52]. Stroke professionals held a similar view to this, expressing a desire for patients to be confident and have increased self-efficacy, “*My patient was a brilliant example of how important self-efficacy is for success in rehabilitation and self-management*” [70].

People with diabetes indicated that decision-making skills were necessary and valued in order to self-manage effectively. Such skills often meant a shift away from a prescriptive model of adherence to professional advice and instead encompassing the development of an ability to make effective decisions. However, this often led to people feeling dismayed and frustrated by interactions with health professionals:

*It’s life and death to me*. *I feel that strongly about it*. *I think that if I hadn’t taken the empowerment [approach] that I probably would be in much worse physical shape*, *and I probably would have had many more complications*, *and I probably would have been killed by some over-zealous intern or whatever defining it as for my own good* [54].

#### 6. Optimal bio-psychosocial health

Optimal bio-psychosocial health describes the collective elements that contribute towards health. Patients (diabetes, colorectal cancer), family (diabetes, stroke) and health professionals (diabetes, stroke), all recognised the achievement of optimal health as an important self-management outcome. This theme was evident in more studies than other themes and was also represented across all stakeholder groups (patients, families and HCPs).

Staying alive and with an acceptable quality of life, was explicitly expressed as a reason for successful self-management by patients, ‘‘*My goal is to keep living*. *I will fix my health care when it interferes with my living*.” (female, diabetes patient [73]) ‘‘[I want to] *stack the odds in my favour*” (male colorectal cancer patient, [50]). For people with diabetes, this meant preventing complications that would affect quality of life, such as losing sight or mobility, “[I fear] *the possibility I might lose my eyesight a little quicker*….” (white female, diabetes patient [74]). Conversely, for people with colorectal cancer, achieving optimal physical health focused around being able to cope with the symptoms of their condition and treatment, and to prevent the reoccurrence of cancer, ‘*‘I do everything I possibly can with hygiene*, *diet*, *all these things to keep me as fit as possible”* (male colorectal cancer patient [67]). For all stakeholders, achieving optimal physical health was thought to be achievable by improving, or maintaining, biomedical indicators of health, such as maintenance of a healthy weight, a balanced diet and exercise, “*The first thing for me*, *was to try and get all the weight back on…I was just eating anything and everything to get my weight on*” (Male colorectal cancer patient, [50]) and “*I feel good when I do walk*” (female, diabetes patient, [75]). Unique to diabetes this also encompassed maintenance of blood glucose levels. Blood glucose control was unanimously valued by diabetic health professionals and often cited as the prime aim of diabetes care, echoing clinical guidelines (e.g. [76, 40]). In the case of ‘poor’ control, blame was often attributed to the patient, as “*the patient had failed to perform the recommended behaviours*” (health care practitioner [48]). Family members valued blood glucose control, with some implementing strategies to assist control “*I often use gradoomthong which is a kind of grass…I boil it with water…and [husband] can drink it every day*. *It helps to reduce his level of blood sugar*” (wife of patient with T2 diabetes, [65]).

For patients (diabetic) performing tasks to achieve physical health appeared to simultaneously improve emotional health “*I keep up with my diet*, *exercise and insulin and I feel good*” (Male T2 diabetes patient, [51]). The interplay between physical and emotional health was recognised by all stakeholders. This was especially so in terms of feeling confident in the ability to self-manage the physical aspects of their condition. For example, going to ‘*diabetes school’* was beneficial, “[it] *puts your mind a rest to go there…*” (Male diabetes patient, [64]). Emotional wellbeing was improved as a result of confidence in ability to self-manage physical health.

For patients, social health was a motivating force behind a need to have good physical health, and a contributing factor to emotional health. There was a need to look after physical health to foster social wellbeing, such as enabling the desire to spend time with family and to look after relatives. One patient reports trying “*to figure out the things that I need to do to stay healthy* …. [to] *stay around a little bit longer so I can take care of my family*” (diabetes patient [73]). Family members of people with stroke agreed that social wellbeing was important. They identified that managing relationships and assistance with communication problems were priorities for self-management support [52].

Achieving optimal health appears to be a result of a complex interplay between three equally important elements, physical, emotional and social health. An important outcome of self-management may therefore be the achievement of optimal biological (physical), psychological, and social health.

## Discussion

This review is the first to investigate the outcomes of self-management that are valued by differing stakeholder groups (patients, families, health professionals and commissioners of services), drawn from international research and from a range of study designs. Our review and synthesis demonstrate different emphases and interpretations of the outcomes that matter between stakeholder groups.

Commissioner / decision-maker / policy maker views are not represented in our review, despite extensive search terms, which were developed with assistance from an information scientist and which attempted to capture this. Such decision makers are responsible for funding services that support self-management, and have a key role to play in the shift in culture and values of health systems towards supporting self-management [77], yet their priorities, in terms of which outcomes they seek, have not yet been clearly articulated in the literature. The extent to which the priorities of commissioners map with the stakeholders they aim to support is unknown, representing a crucial limitation in current understandings and an important area for future research.

We identified that SM outcomes embrace a range of indicators, from knowledge, skills, and bio-psychosocial markers of health, through to ‘being me’ and having positive social networks. There were differences between stakeholders with regard to the outcomes of self-management which were identified (Table 4). Identification of these outcomes does not allow examination of the potential differences between the *value* that different stakeholders place on these outcomes; this is beyond the scope of this review. Our review found that self-management outcomes for patients included a focus on the maintenance and prolonging of health and independence. Patients in our review identified that receiving support for self-management which is meaningful and relevant to the context of their lives is an important outcome. However, services often focus upon process driven outcomes, such as length of stay, or change in blood glucose, without necessarily considering the meaningfulness of this for the patient [78]. Therefore, a successful self-management intervention would demonstrate that patients have received information and support which is tailored and applicable, and not just measure condition knowledge or contact with professionals. Health professionals need to understand this need for tailoring of SM support in their interactions with patients. Having a positive social network was also a necessary SM outcome for patients in accord with recent findings on the illness support work performed by personal communities [79,80]. Patients expressed a desire for support for self-management to be provided from both well-informed, approachable health professionals, who are sensitive to patients as individuals and a supportive network which may include family members and charities.

Despite wide acknowledgment that families play an important part in self-management support [81, 82] few studies explicitly identified families’ perspectives. An important outcome of self-management for families in this review was gaining knowledge about the health condition and how best to help support management, including strategies for coping with emotional issues. Living with a long-term condition, often requires adjustments or behaviour change that impact upon the whole family, such as dietary changes or mood fluctuations. Including families, and evaluating the impact of this, would seem prudent for the future planning and development of self-management support.

In healthcare, there may be an overreliance on the use of biomedical markers as indicators of health care quality and self-management support [83]. In the UK at least, this may be related to the financial incentives associated with the recording and collating of such indicators [84]Qof. Our findings suggest that biomedical outcomes were important to all stakeholders but exist side by side with and may potentially be at tension with patients’ need to ‘be me’, achieve independence and to adapt knowledge to individual contexts. Our review found that indicators of health were not confined to physical aspects of health, but included and were inter-connected with, emotional and social SM outcomes. SM outcome measures therefore need to embrace the full range of bio-psychosocial outcome indicators that are deemed important; at present, many outcome measures focus disproportionately on physical health indicators (see e.g. 27). Where different emphases on self-management outcomes exist, this may result in non-disclosure of behaviours that patients perceive are viewed by professionals as potentially harmful, and thus opportunities to collaboratively discuss ways in which self-management strategies could be safely incorporated into life (the priority for the patient) are potentially missed [85, 86] In addition, measures of LTC monitoring used in clinical practice settings also need to incorporate a holistic focus–in the UK at present, outcome frameworks used for monitoring long term condition management are focused on physical health or process indicators [84].

Our findings indicate that whilst there was consensus around broad outcomes, different stakeholders often appeared to interpret the same outcomes in different ways and emphasised the value of different aspects of those outcomes. This is likely to relate to different stakeholder conceptualisations of self-management and self-management support [87]. How conceptualisations of self-management differ or are similar for the three exemplar conditions warrants further investigation. For some the term ‘self-management’ may imply total independence from health services, whilst for others it incorporates the need for support and appropriate and timely access to that support when circumstance change. Similarly, there may be intra- and inter-stakeholder tensions about the extent to which ‘good’ self-management means strict adherence to best healthcare advice (a perspective closer to that of the HCPs in this review) whilst for others it means adapting advice and modifying adherence in order to live well (a perspective closer to that of patients in this review). Questions therefore exist with regard to how each stakeholder conceptualises and perceives responsibility for self-management [88, 89]. Therefore, research which seeks to understand how stakeholders conceptualise self-management and the desired outcomes of differing approaches is required to further broaden the knowledge base.

The majority of evidence included in the synthesis was generated from patient and health professional perspectives, indicating a need for the perspectives of other stakeholder groups to be more thoroughly investigated. For the purposes of this review, three conditions were selected for potential differences in self-management foci, however the majority of included evidence was generated from diabetes (32 of 40 papers). Other long-term conditions may feasibly generate different outcomes of value, which should be considered as the self-management knowledge base develops. Given that an aim of this review was to prioritise studies which explicitly sought different stakeholders’ perspectives, it is unsurprising that the majority of evidence originated from qualitative findings. The majority of potential research identified was excluded (265 papers, 87%) because there was no rationale stated for the outcomes selected. Feasibly, authors of the identified studies had acceptable justification for the selection of outcome measures but overlooked specifying this, narrowing the potential evidence and representing a possible limitation of this synthesis. In future trials of self-management, and as set out by others, researchers should clearly specify the theoretical link between the measure and self-management *and*, to be meaningful to all stakeholders, seek to select outcome measures that capture outcomes generated from and/or are relevant to stakeholder perspectives [90, 91].

This review and synthesis is not without some limitations. No included study explicitly focused upon the outcomes of self-management *per se*. Additional evidence may be available from studies that did not explicitly specify they concerned self-management, but nevertheless have a similar focus, for example on health behaviour change or secondary prevention. A lack of evidence for the importance of an outcome is not necessarily a reflection of a lack of importance to the different stakeholders but, rather, may reflect a lack of previous research explicitly seeking their views on this topic. Further research is therefore required to build on these early findings from the existing literature to identify which outcomes of self-management are important from the perspectives of differing stakeholders.

The absence of a universally recognised quality criteria tool for studies drawn from mixed research designs is a limitation. Although studies were not excluded on the basis of quality, quality judgements from well conceptualised and validated assessment tools would add rigour to interpretation of these findings. Arguably, the methodological quality of most of the included studies was weak (Table 2). Overarching reasons for weak quality ratings included a lack of quotations that were identifiable to differing individuals, an absence of analysis methodology and limited sampling.

## Conclusions

This review identified which SM outcomes have been articulated by different groups of stakeholders. Although the quality of evidence is limited, we identified that SM outcomes should embrace a range of indicators, from knowledge, skills, and bio-psychosocial markers of health through to positive social networks. An important subject that ran through the findings was the need for SM outcomes to be applied to patients’ everyday lives. Few studies have sought to explicitly identify the desired outcomes of self-management from the perspectives of stakeholders, and commissioner perspectives are currently absent. Further research is required to identify both the conceptual underpinnings of self-management from each stakeholder perspective and which self-management outcomes they consider important to guide the commissioning and design of future self-management services.

## Supporting Information

S1 Fig (PDF)

S2 Fig (PDF)
