# Supplementary material for: Self-Management and Self-Management Support Outcomes: A Systematic Review and Mixed Research Synthesis of Stakeholder Views
Source: PLoS One. 2015 Jul 10;10(7):e0130990. doi: 10.1371/journal.pone.0130990 (PMC4498685; doi:10.1371/journal.pone.0130990)
Supplement: S1 Fig — (PDF) [file pone.0130990.s001.pdf]

S1 Figure - Search strategy

The 9 datasets that were used for searching the databases are listed below.

The databases were chosen as they provided a systematic overview of the maximum number of journals worldwide to ensure a comprehensive search of the publications indexed. We conducted the searches during the period 10th December 2013 - 31st January 2014.

Datasets were used to search the following resources:

EBSCO (PsychInfo, CINAHL, Medline)

Health Care databases via Evidence Search NHS. (HMIC, BNI, AMED, Health Business Elite, EMBASE,)

Web of Science

Delphis (University of Southampton Discovery System)

Cochrane Library.

We used a combination of free text and subject headings using Title and Abstract as a limiter within our datasets as we were using multiple databases each with their own thesauri and this ensured maximum specificity without too many inappropriate references.

1. TI patient* OR AB patient*
2. TI (commission* or stakeholder* or "Chronic Disease Economics" or "resource allocation" or "decision maker*" or "healthcare service provider" or "health care service provider") OR AB (commission* or stakeholder* or "Chronic Disease Economics" or "resource allocation" or "decision maker*"or "healthcare service provider*" or "health care service provider")
3. TI ("diabetes mellitus" or diabet* or "diabetic patient*) OR AB ("diabetes mellitus" or diabet* or "diabetic patient*")
4. TI (outcome* or "outcome measur*" or "outcome assessment*" or intervention* or evaluat* or result* or finding*) OR AB (outcome* or "outcome measur*" or "outcome assessment*" or intervention* or evaluat* or result* or finding*)
5. TI (stroke* or "stroke patient*" or CVA or "cerebrovascular accident" or TIA or "transient ischemic attack") OR AB (stroke* or "stroke patient*" or CVA or "cerebrovascular accident" or TIA or "transcient ischemic attack")
6. TI (famil* or caregiver* or "care giver*" or "caregiver burden" or partner* or spouse or friend*) OR AB (famil* or caregiver* or "care giver*" or "caregiver burden" or partner* or spouse or friend*)
7. TI ("self care" or "self manag*" or "self monit$r*" or "Chronic Disease Management") OR AB ("self care" or "self manag*"or "self monit$r*" or "Chronic Disease Management")
8. TI ("bowel cancer" or "cancer of the bowel" or "colorectal cancer" or "rectal cancer" or "bowel carcinoma" or "anal cancer") OR AB ("bowel cancer" or "cancer of the bowel" or "colorectal cancer" or "rectal cancer" or "anal cancer" or "bowel carcinoma")
9. TI ("health personnel" or "healthcare personnel" or "health care personnel" or "healthcare professional" or "health care professional”) OR AB ("health personnel" or "healthcare personnel" or "health care personnel" or "healthcare professional" or "health care professional”)

Database	Interface	Specific Date (if known)
CINAHL Plus	EBSCOhost	
Medline	EBSCOhost	
PsychInfo	EBSCOhost	
HMIC	NHS Evidence	15.01.14
BNI	NHS Evidence	15.01.14
AMED	NHS Evidence	15.01.14
Health Business Elite	NHS Evidence	15.01.14
EMBASE	NHS Evidence	15.01.14
WoS	Web of Science	23.01.14
Medline (1950-)	Web of Science	23.01.14
JCR (Science and Social Science editions)	Web of Science	23.01.14
BIOSIS Citation Index (2009-2011)	Web of Science	23.01.14
BIOSIS Citation Index Current Data 2012	Web of Science	23.01.14
Inspec	Web of Science	23.01.14
Holdings	Web of Science	23.01.14
OpenURL Resolver	Web of Science	23.01.14
JSTOR	Web of Science	23.01.14
	Delphis	
EndNote Basic	Web of Science	23.01.14
IP Authentication-Federated Access	Web of Science	23.01.14
Shibboleth	Web of Science	23.01.14
WoK 2013-16	Web of Science	23.01.14
JCR 2013-16	Web of Science	23.01.14
