# Supplementary material for: Self-Management and Self-Management Support Outcomes: A Systematic Review and Mixed Research Synthesis of Stakeholder Views
Source: PLoS One. 2015 Jul 10;10(7):e0130990. doi: 10.1371/journal.pone.0130990 (PMC4498685; doi:10.1371/journal.pone.0130990)
Supplement: S2 Fig — (PDF) [file pone.0130990.s002.pdf]

S2 Fig. Criteria used to appraise study quality

Adapted from: Rees, R., Oliver, K., Woodman, J., & Thomas, J. (2009). *Children' s views about obesity, body size, shape and weight: A systematic review*. *EPPI Centre:* London.

Guidance from: Hannes, K. (2011) Chapter 4: Critical appraisal of qualitative research. In: Noyes J, Booth A, Hannes K, Harden A, Harris J, Lewin S, Lockwood C (editors), *Supplementary Guidance for Inclusion of Qualitative Research in Cochrane Systematic Reviews of Interventions*. Version 1 (updated August 2011). Cochrane Collaboration Qualitative Methods Group.

Tong, A. et al. (2007). Consolidated criteria for reporting qualitative research (COREQ): a 32-item checklist for interviews and focus groups. *International Journal for Quality in Health Care*; 19 (6), pp. 349–357.

http://ph.cochrane.org/sites/ph.cochrane.org/files/uploads/Unit_Eight.pdf

**1: Was the sample appropriate for the research question?**<br><br>**Consider whether:**<br><br>*****the sampling strategy was appropriate to the question posed in the study (was the strategy well reasoned and justified? Was there selection bias?)<br><br>*attempts were made to obtain a diverse sample of the population in question (who might have been excluded?)<br><br>*characteristics of the sample critical to the understanding of the study context and findings were presented. (e.g. socio-demographics, characteristics relevant to the context of the study, etc.).	**Yes, a fairly thorough attempt was made (3)**<br><br>**Yes several steps were taken (2)**<br><br>**Yes a few steps were taken (1)**<br><br>**No, not at all/not stated/can't tell (0)**
**2: Were steps taken to increase rigour/reduce bias in data collection?**<br><br>**Consider whether:**<br><br>*****data collection tools were piloted<br><br>*data collection methods were congruent with the research methodology and research question/ aim;<br><br>*steps were taken to ensure that all participants were able and willing to contribute (e.g. processes for consent, barriers);<br><br>*nature of the relationship between the researcher and participants (the relationship and extent of interaction between the researcher and their participants should be described) and any reflexivity<br><br>*Qual only- data collection was comprehensive, flexible and/ or sensitive enough to provide a complete and/ or vivid and rich description of people's perspectives and experiences (e.g. did the researchers spend sufficient time at the site / with participants? Were they sensitive to interviewing people with fatigue problems? Was more than one method of data collection used?)<br><br>Quan only - outcomes have been measured with valid and reliable instruments; assessors were blinded or double-blinded as appropriate; populations were similar prior to any treatment effect/intervention; were all treated using the same procedure	**Yes a fairly thorough attempt was made (3)**<br><br>**Yes several steps were taken (2)**<br><br>**Yes minimal few steps were taken (1)**<br><br>**No not at all/ Not stated/ can't tell (0)**

**3: Were steps taken to increase rigor/reduce bias in the analysis of the data?**<br><br>**Consider whether:**<br><br>*data analysis methods were systematic and appropriate to research question/aim (e.g. was a method described/ can a method be discerned?);<br><br>*****data analysis methods were congruent with the research methodology;<br><br>*Qual only - diversity in perspectives were explored; the analysis was balanced in the extent to which it was guided by preconceptions or by the data; the analysis sought to rule out alternative explanations for findings (e.g. searching for negative cases/ exceptions, feeding back preliminary results to participants, asking a colleague to review the data); reflexivity (if the researcher critically examined their own role, potential bias and influence during data analyses).<br><br>*Quan only- the sample was powered for statistical significance; a sample size calculation is stated; the fidelity of any intervention/treatment is clear; confounding factors been removed from analysis?	**Yes a fairly thorough attempt was made (3)**<br><br>**Yes several steps were taken (2)**<br><br>**Yes minimal few steps were taken (1)**<br><br>**No not at all/ Not stated/ can't tell (0)**
**4: Were the conclusions of the study supported by the data?**<br><br>**Consider whether:**<br><br>Qual only: enough data are presented to show how the authors arrived at their findings; the data presented fit the interpretation/ support claims about patterns in the data; the data presented illuminate /illustrate the findings; quotes are numbered or otherwise identified and the reader can see that they don't just come from one or two people.<br><br>Quan only: consider whether the limitations of the study are stated; are any limitations of the study not accounted for; are the design and methods of the study sufficiently flawed to make the results unreliable; do any conclusions reflect the results of statistical analyses.	**Good grounding/support (2)**<br><br>**Fair grounding/support (1)**<br><br>**Limited grounding/support (0)**
**5: Quan only: How generalisable are the results of the study?**<br><br>*Are participants in the study similar enough to wider populations? If not, how do they differ?<br><br>*Are any benefits worth the harms and costs?<br><br>*Are all relevant outcomes reported on? Is there other information that should have been reported?	**Limited generalisability (0)**<br><br>**Fair generalisability (1)**<br><br>**Good generalisability (2)**<br><br>**Excellent generalisability (3)**
**6: Qual only: To what extent does the study privilege the perspectives and experiences of stakeholders?**<br><br>**Consider:**	

***** whether there were open –ended response options;<br><br>*whether stakeholders were involved in designing the research;<br><br>*whether there was a balance between the use of an a priori coding framework and induction in the analysis;<br><br>* the position of the researchers: 1) Did they consider it important to listen to the perspectives of participants? 2) Are the researcher's experiences and assumptions outlined? (e.g. if researchers explored their background, epistemological basis and perception of research at inception);<br><br>*whether steps were taken to ensure confidentiality and putting stakeholders at ease.	**Not at all (0)**<br><br>**A little (1)**<br><br>**Somewhat (2)**<br><br>**A lot (3)**
**7: Overall what weight would you assign to this study in terms of the reliability/trustworthiness of its findings?**<br><br>**Guidance:**<br><br>Think mainly about your answers you have given to question 1 to 6 above	**Low (1)**<br><br>**Medium (2)**<br><br>**High (3)**
**Total (0-20)**	
**8: What weight would you assign to this study in terms of the usefulness of findings for this review?**<br><br>**Guidance:**<br><br>Consider:<br><br>**1. Research which focusses on the expressed views of stakeholders with regard to SM outcomes (5)**<br><br>**2. Research about experiences of SM with stakeholders which makes reference to SM outcomes (4)**<br><br>**3. SM interventions which imply that the aims of the intervention, or outcome measures selected, were directed by stakeholder input (3)**<br><br>**4. SM interventions which state the reasons for the selection of any outcome measures or aims of the intervention (2)**<br><br>**5. SM interventions which identify the expected outcomes of the intervention (1)**	**High (4/5)**<br><br>**Medium (3)**<br><br>**Low (1/2)**
**Total (1-5)**	
